# Progressive Grey Matter Volume Changes in Patients with Schizophrenia over 6 Weeks of Antipsychotic Treatment and Their Relationship to Clinical Improvement

**DOI:** 10.1007/s12264-018-0234-6

**Published:** 2018-05-19

**Authors:** Xiao Zhang, Yuyanan Zhang, Jinmin Liao, Sisi Jiang, Jun Yan, Weihua Yue, Dai Zhang, Hao Yan

**Affiliations:** 1 Peking University Sixth Hospital/Institute of Mental Health, Beijing, 100191 China; 2 Key Laboratory of Mental Health, Ministry of Health (Peking University) and National Clinical Research Center for Mental Disorders (Peking University Sixth Hospital), Beijing, 100191 China; 3 Peking-Tsinghua Joint Center for Life Sciences and PKU IDG/McGovern Institute for Brain Research, Peking University, Beijing, 100871 China

**Keywords:** Schizophrenia, Grey matter volume, Rostral medial frontal cortex, Treatment response, Biomarker

## Abstract

Cross-sectional and longitudinal studies have identified widespread and progressive grey matter volume (GMV) reductions in schizophrenia, especially in the frontal lobe. In this study, we found a progressive GMV decrease in the rostral medial frontal cortex (rMFC, including the anterior cingulate cortex) in the patient group during a 6-week follow-up of 40 patients with schizophrenia and 31 healthy controls well-matched for age, gender, and education. The higher baseline GMV in the rMFC predicted better improvement in the positive score on the Positive and Negative Syndrome Scale (PANSS), and this might be related to the improved reality-monitoring. Besides, a higher baseline GMV in the posterior rMFC predicted better remission of general symptoms, and a lesser GMV reduction in this region was correlated with better remission of negative symptoms, probably associated with ameliorated self-referential processing and social cognition. Besides, a shorter disease course and higher educational level contributed to better improvement in the general psychopathological PANSS score, and a family history was negatively associated with improvement of the negative and total PANSS scores. These phenomena might be important for understanding the neuropathological mechanisms underlying the symptoms of schizophrenia and for making clinical decisions.

## Introduction

Schizophrenia is one of the most serious and disabling psychiatric disorders with ~1% prevalence across the world [1]. It is a complex syndrome with a heterogeneous combination of symptoms, which can be divided into positive, negative, and cognitive categories [2]. Widespread changes in grey matter volume (GMV) and progressive GMV reduction have been repeatedly reported in cross-sectional [3] and longitudinal [4] studies in both first-episode and chronic schizophrenic patients. In first-episode patients, the volumetric deficits are subtle and mainly located in parts of the frontal, parietal, and middle temporal cortices [5, 6], and in chronic patients, evidence from a systematic review and meta-analysis has demonstrated that brain tissue decreases are continuously progressive; the annualized percentage GMV reduction in chronic schizophrenia is ~0.5% compared to healthy individuals, the frontal and temporal areas being the most affected regions [7, 8].

Although it has been suggested that the loss in schizophrenia is a combination of early neurodevelopmental problems as well as disease progression [9], the factors associated with the progressive brain changes in schizophrenia are still unclear. Previous meta-analyses have indicated that higher cumulative exposure to antipsychotics over time is associated with a greater longitudinal reduction of GMV in patients with schizophrenia [9, 10]. Yet some studies have shown that antipsychotic treatment has an incremental effect on the caudate, thalamus, and parts of the frontal and temporal parietal lobule, with a significant grey matter reduction in parts of the frontal gyrus [11, 12]. Another factor that may affect GMV is the duration of illness, i.e., a greater GMV reduction is associated with a longer duration, and this has been validated by many studies [13–15].

Current evidence has identified an effect of antipsychotic exposure on progressive GMV changes, which raises the possibility of establishing a relationship between GMV and the efficacy of medication. Several studies have focused on this topic, but the findings are inconsistent. Some have reported that reductions in total and regional GMV, such as in prefrontal, middle frontal, hippocampal, and caudate volumes, are associated with a relatively poor response to treatment [16, 17]. A voxel-wise volume study on first-episode drug-naive patients with schizophrenia found a significant GMV increase in the right putamen after 6 weeks of antipsychotic treatment, and this increase was associated with an improvement in positive symptoms [18]. However, in a very recent study, although cortical volume reductions occurred in drug-naive patients during their first year of treatment, they showed no significant association with changes in the symptoms, treatment-related side-effects, age, gender, and duration of untreated psychosis [19].

Besides GMV changes, some studies have also investigated whether the baseline GMV, a better and practical predictor, is associated with the treatment response in schizophrenia. In a computed tomography study, a smaller prefrontal sulcal prominence was found to correlate with a better clozapine response in treatment-resistant patients [20]. The baseline temporal GMV was positively correlated with an improvement in positive symptoms, whereas a high baseline GMV in the dorsolateral prefrontal cortex was associated with an improvement of the negative symptoms in treatment-resistant patients [21]. Only poor-outcome patients, and not good-outcome patients, displayed a significantly smaller frontal GMV [17, 22]. Further, a study also showed different interactive effects between brain structure and the treatment responses to clozapine and haloperidol; the right prefrontal cortex GMV was positively correlated with changes in the total brief psychiatric rating scale score in the haloperidol group, while the opposite was found in the clozapine group [22].

In the present study, using a prospective study design, we investigated the progressive GMV changes in schizophrenia over 6 weeks of antipsychotic monotherapy and the relationship of baseline and follow-up GMVs to the antipsychotic efficacy.

## Participants and Methods

### Participants

This study was approved by the Medical Ethics Committee of Peking University Sixth Hospital. Patients were recruited from the Peking University Sixth Hospital and they all provided written informed consent after a description of the study. All patients were assessed and diagnosed as suffering from schizophrenia or schizophreniform psychosis by trained psychiatrists using the Structured Clinical Interview for DSM-IV-TR Axis I Disorders (SCID-I, patient edition). All patients recruited first as having schizophreniform psychosis were finally diagnosed with schizophrenia after being followed up for at least 6 months. Patients with neurological disorders, a history of serious medical illness, substance dependence, pregnancy, or those treated with electroconvulsive therapy within the last 6 months and a diagnosis of any other Axis I disorder were excluded. Healthy controls (HCs) were recruited from the local community through advertisement and screened using the SCID-I (non-patient edition). HCs had no life-time history of psychotic illness and no family history of psychosis. HCs had exclusion criteria similar to patients and were well matched to the patient group for age, gender, and educational level. All participants were Han Chinese and right-handed.

All participants underwent magnetic resonance image (MRI) scanning and completed clinical assessments at baseline. After 6 weeks, 41 patients and 31 HCs were included in the follow-up procedure that included clinical assessments and MRI scanning. The symptom severity in all patients was assessed by trained and experienced psychiatrists using the Positive and Negative Syndrome Scale (PANSS), and the change of PANSS score from baseline to 6 weeks was used as the index of medication efficacy. Strict quality control was performed to ensure the completeness of the demographic and treatment information and that the MRI data were of high quality. After careful examination, 40 patients and 31 HCs were finally included in the analysis (Table 1). The clinical characteristics of patients with schizophrenia during the 6 weeks of treatment are listed in Table 2. In the current study, 90% of patients were receiving antipsychotic treatment without antidepressants or mood stabilizers. The medication dosage at baseline and follow-up was converted to chlorpromazine equivalents (CPZ-eq) (Table 2).

**Table 1:** Baseline demographic characteristics of schizophrenic patients and healthy controls.

	Schizophrenic patients	Healthy controls	*t* or χ^2^	*P* value
Gender (male/female)	21/19	18/13	0.218	0.640
Age (years)	27.7 ± 7.7	26.2 ± 6.8	0.831	0.409
Education (years)	13.5 ± 3.2	13.2 ± 2.8	0.500	0.619
Total gray matter volume (cm^3^)	706.4 ± 66.7	726.5 ± 56.5	−1.342	0.184

Data are given as mean ± standard deviation. *P*-values refer to the independent-sample *t*-test (parametric data) and the *χ*^2^ test (categorical data).

**Table 2:** Clinical characteristics of schizophrenic patients during 6 weeks of treatment.

	Baseline	Follow-up^#^	*t* or χ^2^	*P* value
Illness duration (months)	53.5 ± 53.3	–	–	–
First/relapse (No.)	18/22	–	–	–
Paranoid/others (No.)	38/2	–	–	–
Onset age (years)	23.7 ± 6.8	–	–	–
Times of hospitalization	1.5 ± 1.5	–	–	–
Family history (Yes/No)	14/26	–	–	–
CPZ-eq (mg/day)*	446.3 ± 223.1	556.9 ± 309.9	−2.136	0.039
Positive PANSS score	24.3 ± 4.3	15.1 ± 4.5	10.811	<0.001
Negative PANSS score	20.2 ± 5.6	17.1 ± 6.1	4.750	<0.001
General psychopathological PANSS score	35.7 ± 6.0	27.2 ± 5.9	10.364	<0.001
Total PANSS score	79.8 ± 9.8	59.4 ± 13.0	12.504	<0.001
Total gray matter volume (cm^3^)	706.4 ± 66.7	701.3 ± 64.8	2.619	0.012

Data are given as mean ± standard deviation. *P*-values refer to paired-samples *t*-test. CPZ-eq, chlorpromazine equivalent dose; PANSS, Positive and Negative Syndrome Scale.

*At baseline, 37 patients were taking antipsychotic monotherapy (Aripiprazole, 3; Amisulpride, 3; Blonanserin, 4; Haloperidol, 1; Iloperidone, 1; Olanzapine, 10; Paliperidone, 3; Quetiapine, 1; Risperidone, 11; Aripiprazole + Risperidone, 1; Haloperidol + Risperidone, 2). At follow-up, 39 patients were taking antipsychotic monotherapy (Aripiprazole, 4; Amisulpride, 4; Blonanserin, 2; Olanzapine, 12; Paliperidone, 2; Quetiapine, 2; Risperidone, 13; Amisulpride + Paliperidone, 1).

^#^Follow-up PANSS scores were missing for two patients.

### MRI Data Acquisition and Processing

All participants were scanned on a 3.0 T Siemens Trio MR scanner (Siemens Medical Systems, Erlangen, Germany) at the Third Hospital, Peking University. Before scanning, all participants were instructed to move as little as possible. Foam pads were used to minimize head motion. T1-weighted high-resolution structural images were acquired in a sagittal orientation using a 3D-MPRAGE sequence with the following parameters: repetition time = 2350 ms, echo time = 3.44 ms, field of view = 256 × 256 mm^2^, slice thickness/gap = 1.0/0 mm, acquisition voxel size = 1 × 1 × 1 mm^3^, flip angle = 12°, 192 contiguous sagittal slices. Quality control to exclude potential imaging artifacts was carried out independently by two researchers.

The structural images were processed with DPABI [23], a MatLab toolbox that calls for SPM12 (http://www.fil.ion.ucl.ac.uk/spm) and DARTEL was used [24] to perform VBM (voxel-based morphometry) analysis with default parameters. DARTEL is believed to have improved segmentation results [25]. Image processing included: (1) Transforming structural images into NIFTI format. (2) Reorienting the structural images iteratively so that the millimeter coordinates of the anterior commissure matched the origin. (3) Segmenting T1-weighted MR images into grey matter, white matter, and cerebrospinal fluid using “Segment” in SPM12. (4) Computing transformations from individual native space to MNI (Montreal Neurological Institute) space for registration, normalization, and modulation using DARTEL. (5) Smoothing the segmented, normalized, and modulated GM images with an 8-mm full-width-at-half-maximum isotropic Gaussian kernel.

### Statistical Analysis

To assess the progressive changes of GMV in patients with schizophrenia, we compared their data at baseline and follow-up in two directions using a paired *t*-test model in statistical parametric mapping (SPM) with total GMV as covariates. Significant effects were those that survived a *P* < 0.05 whole-brain family-wise error (FWE) correction with a cluster size >10 to remove possible false-positives. A similar method was used in HCs.

We quantified the treatment response as the percentage changes in PANSS scores (positive, negative, general psychopathological, and total) from baseline to week 6 [26]. Given the longitudinal design, we focused on the biological importance of progressive GMV changes. The baseline and follow-up GMVs of the peak voxels were extracted for further analysis. For the peak coordinates in the anterior and posterior rMFC, we did multilinear regression with the rate of PANSS change as the dependent variable, with gender, education, onset age, duration of illness, first-episode or relapse, times of hospitalization, family history, baseline CPZ-eq dose, follow-up CPZ-eq dose, baseline total GMV, total GMV change, baseline GMV of peak voxel and GMV change of peak voxel as the independent variables. We did not include age in this model since it is highly correlated with onset age. These models allowed us to explore the potential role of each peak voxel after excluding the potential influences of all other variables, especially total GMV. However, considering the relatively high correlation between the total GMV and regional GMV both at baseline (anterior rMFC: *r *= 0.736, *P* < 0.001; posterior rMFC: *r *= 0.760, *P* < 0.001) and during the period (anterior rMFC: *r* = 0.802, *P* < 0.001; posterior rMFC: *r *= 0.760, *P* < 0.001), the influence of total GMV was explored in another multilinear model (total GMV model) with the rate of PANSS change as the dependent variable, with the parameters noted above, except for regional GMV items, as independent variables. We used the Statistical Package for the Social Sciences 20.0 (IBM SPSS, Chicago, IL) to conduct these regression models, with all variables included in the three models passing the multicollinearity test (two participants missed follow-up PANSS scores and were excluded from these regression models).

To validate the relationship between baseline rMFC and the rate of PANSS change found from the multilinear regression model (see Results, effects of GMV on the rate of PANSS change), we did an exploratory analysis in SPM using whole-brain voxel-wise regression on baseline GMV with PANSS change rate as the dependent variable. The covariates were gender, education, onset age, duration of illness, first-episode or relapse, times of hospitalization, family history, baseline CPZ-eq dose, follow-up CPZ-eq dose, baseline total GMV, and total GMV change.

## Results

### Demographics and Clinical Characteristics

There were no significant differences in gender distribution, age, education, and total GMV between patients with schizophrenia and HCs (Table 1). According to the PANSS scores, patients with schizophrenia were moderately-to-severely impaired at baseline and remarkably relieved after 6 weeks of treatment (Table 2).

### Longitudinal Brain Changes in Patients

**Fig. 1:**
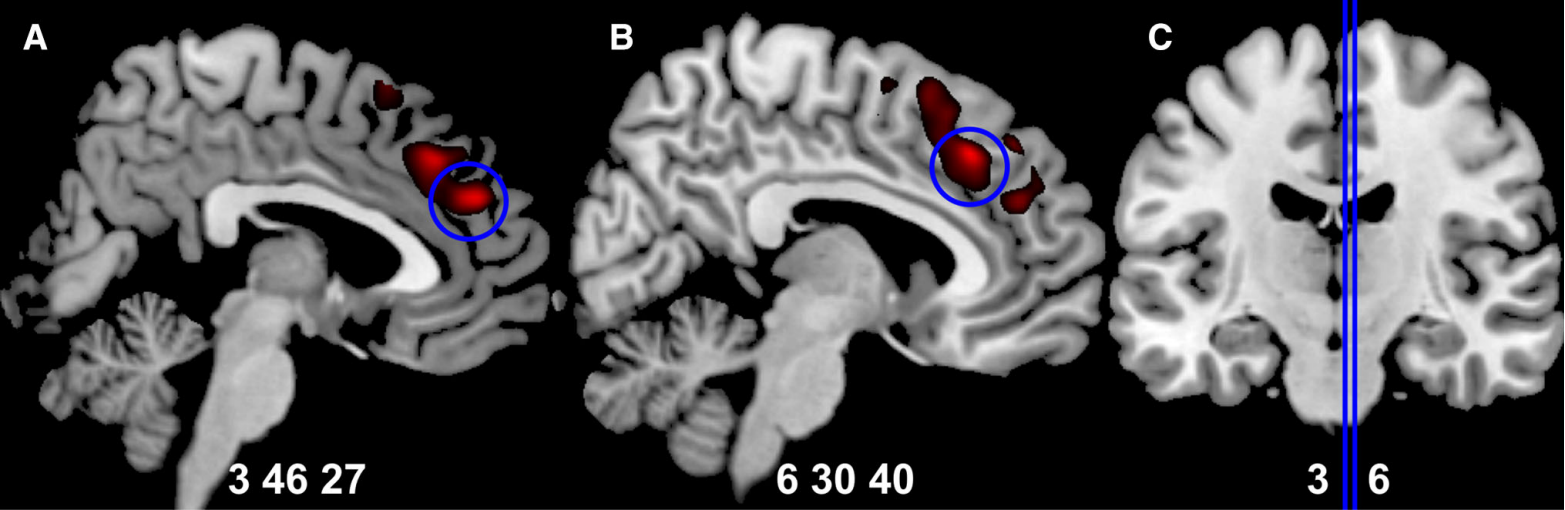
GMV reduction after six weeks of treatment in patients with schizophrenia (shown at *P* < 0.001 uncorrected). **A** Regional GMV reduction at the anterior rMFC (including rostral anterior cingulate cortex) (circled peak coordinates = [3, 26, 45], *t *= 5.96, cluster size = 12, *P *= 0.016 FWE corrected). **B** Regional GMV reduction at the posterior rMFC (including dorsal anterior cingulate cortex) (circled peak coordinates = [6, 29, 39], *t* = 6.22, cluster size = 19, *P* = 0.008 FWE corrected). **C** Sagittal slices in panels **A** and **B**.

After antipsychotic treatment for 6 weeks, the total GMV was statistically decreased (Table 2) while this effect was not found in the HCs (726.5 ± 56.5 *versus* 727.0 ± 58.8 cm^3^, *P* = 0.804). As for the regional findings (Fig. 1, *P* < 0.05, FWE corrected, *k *> 10), patients exhibited reduced regional GMV in the rMFC (in which, for convenience, we included the anterior cingulate cortex). The first peak (x = 3, y = 46, z = 27, T = 6.22) was at the anterior rMFC (including the rostral anterior cingulate cortex). The second peak (x = 6, y = 30, z = 40, T = 5.96) was at the posterior rMFC (including the dorsal anterior cingulate cortex). No GMV increase was observed after 6 weeks of treatment. Besides, there were no significant changes of GMV in HCs after 6 weeks.

### Effects of Demographic and Clinical Factors on the Rate of PANSS Change

In all models (Table 3), we found that the family history negatively affected the improvement of the negative and total PANSS scores; and a shorter disease course predicted a larger percentage change in the general psychopathological PANSS score. In the model of the posterior rMFC (Table 3), we further found that a higher educational level predicted a larger percentage change in the general psychopathological PANSS score.

**Table 3:** Regression analysis of the rate of PANSS change with the peak coordinates of GMV, total GMV, and other potential variables (standardized coefficients and standard error).

Model	Variables	Positive CR		Negative CR		General psychopathological CR		Total CR	
		Coefficients	SE	Coefficients	SE	Coefficients	SE	Coefficients	SE
Posterior rMFC GMV Model [6 30 40]	Sex	0.271	0.247	−0.080	0.262	0.136	0.238	0.172	0.223
	Education	0.097	0.222	0.341	0.236	0.515*	0.214*	0.415	0.201
	Onset Age	−0.244	0.210	−0.057	0.223	−0.379	0.202	−0.277	0.190
	Course	0.046	0.267	−0.073	0.284	−0.649*	0.257*	−0.360	0.242
	First/Relapse	−0.206	0.220	0.235	0.234	−0.076	0.212	−0.051	0.199
	Baseline CPZ-eq	−0.245	0.214	−0.063	0.228	−0.018	0.206	−0.096	0.194
	Follow-up CPZ-eq	−0.243	0.179	0.070	0.191	−0.079	0.173	−0.110	0.162
	Times of hospitalization	−0.106	0.276	0.056	0.293	0.270	0.265	0.059	0.250
	Family History	−0.305	0.170	−0.384*	0.180*	−0.170	0.163	−0.331*	0.154*
	Baseline TGMV	−0.496	0.320	−0.165	0.340	−0.749*	0.308*	−0.663*	0.290*
	TGMV Change	0.249	0.256	0.666*	0.272*	0.069	0.246	0.264	0.232
	Baseline RGMV	0.924**	0.247**	0.085	0.263	0.720**	0.238**	0.759**	0.224**
	RGMV Change	−0.353	0.280	−0.748*	0.298*	−0.357	0.270	−0.434	0.254
Anterior rMFC GMV Model [3 46 27]	Sex	0.296	0.281	−0.375	0.297	0.062	0.284	0.107	0.266
	Education	−0.055	0.236	0.198	0.249	0.382	0.238	0.268	0.223
	Onset Age	−0.098	0.225	0.085	0.238	−0.274	0.228	−0.144	0.213
	Course	0.108	0.298	−0.121	0.315	−0.648*	0.301*	−0.335	0.282
	First/Relapse	−0.113	0.244	0.270	0.258	0.062	0.246	0.055	0.230
	Baseline CPZ-eq	−0.178	0.234	−0.038	0.248	0.057	0.237	−0.033	0.222
	Follow-up CPZ-eq	−0.177	0.210	0.135	0.222	0.030	0.212	−0.022	0.198
	Times of hospitalization	−0.267	0.303	−0.003	0.320	0.175	0.306	−0.071	0.287
	Family History	−0.345	0.185	−0.443*	0.196*	−0.205	0.187	−0.373*	0.175*
	Baseline TGMV	−0.302	0.344	−0.222	0.363	−0.488	0.347	−0.494	0.325
	TGMV Change	−0.086	0.354	0.426	0.374	−0.095	0.357	0.010	0.334
	Baseline RGMV	0.621*	0.253*	−0.142	0.267	0.245	0.255	0.419	0.239
	RGMV Change	0.229	0.363	−0.350	0.383	−0.009	0.366	0.038	0.343
TGMV Model	Sex	0.182	0.286	−0.263	0.273	0.046	0.259	0.064	0.253
	Education	−0.072	0.255	0.177	0.243	0.367	0.230	0.246	0.225
	Onset Age	−0.133	0.236	0.147	0.224	−0.270	0.213	−0.146	0.208
	Course	0.012	0.322	−0.062	0.307	−0.674*	0.291*	−0.385	0.284
	First/Relapse	0.025	0.251	0.172	0.239	0.094	0.227	0.122	0.222
	Baseline CPZ-eq	−0.116	0.255	−0.050	0.243	0.082	0.230	0.010	0.225
	Follow-up CPZ-eq	−0.079	0.209	0.039	0.199	0.044	0.188	0.015	0.184
	Times of hospitalization	−0.175	0.330	−0.039	0.314	0.206	0.298	−0.015	0.292
	Family History	−0.343	0.203	−0.452*	0.193*	−0.207	0.183	−0.376*	0.179*
	Baseline TGMV	0.051	0.333	−0.277	0.317	−0.340	0.301	−0.245	0.294
	TGMV Change	0.103	0.196	0.133	0.187	−0.104	0.177	0.039	0.173

*P < 0.05, **P < 0.01. CR, rate of PANSS change; CPZ-eq, chlorpromazine equivalent dose; TGMV, total grey matter volume; RGMV, regional grey matter volume; SE, standard error.

### Effects of GMV on the Rate of PANSS Change

**Fig. 2:**
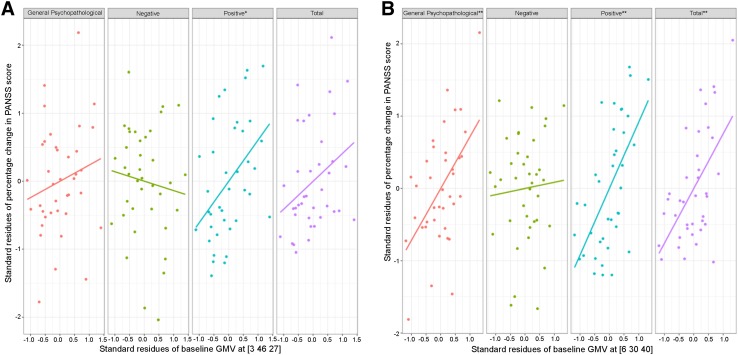
Relationship between baseline regional GMV and the percentage changes of PANSS scores in the multilinear regression model after controlling for potential variables. **A** Baseline anterior rMFC GMV at [3 46 27] positively influenced clinical improvement of the positive PANSS score. **B** Baseline posterior rMFC GMV at [6 30 40] positively influenced clinical improvement of the general psychopathological, positive, and total PANSS scores.

**Fig. 3:**
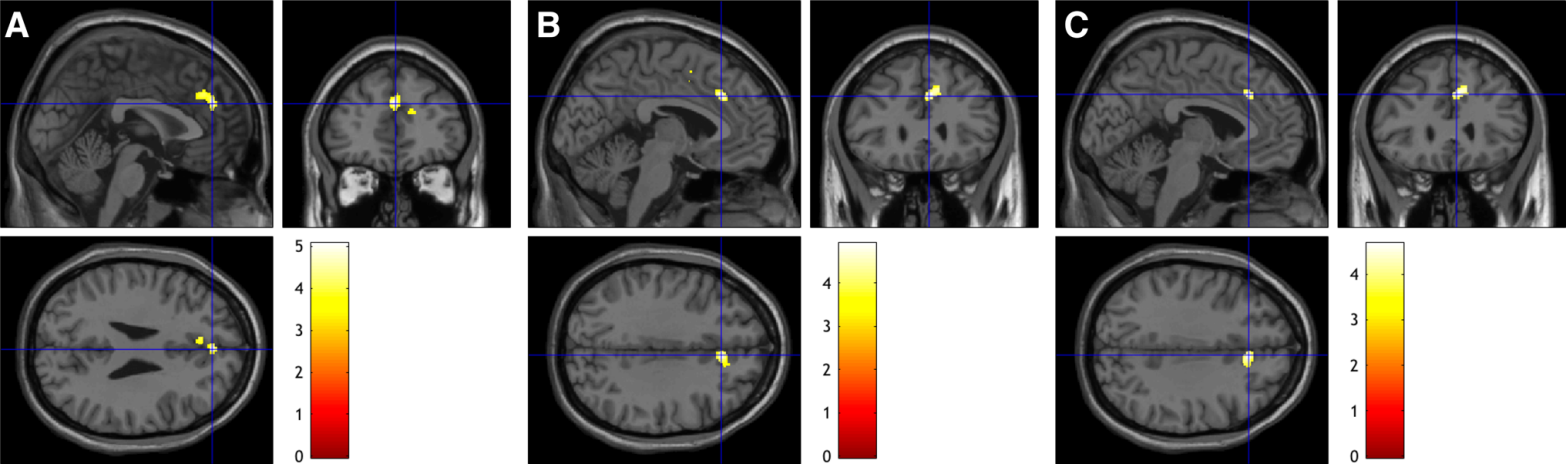
Relationships between the percentage changes of positive, general, and total PANSS scores and the baseline rMFC GMV in whole-brain voxel-wise regression analysis (*P* < 0.001, uncorrected). **A** Positive correlation between the rate of change of positive PANSS score and baseline GMV (cluster size = 638; peak coordinates = [−2 44 27]; *T *= 4.95). **B** Positive correlation between the rate of change of general psychopathological PANSS score and baseline GMV (cluster size = 259; peak coordinates = [3 28 33], *T* = 4.91). **C** Positive correlation between the rate of change of total PANSS score and baseline GMV (cluster size = 225; peak coordinates = [3, 28, 34]; *T* = 4.67).

**Fig. 4:**
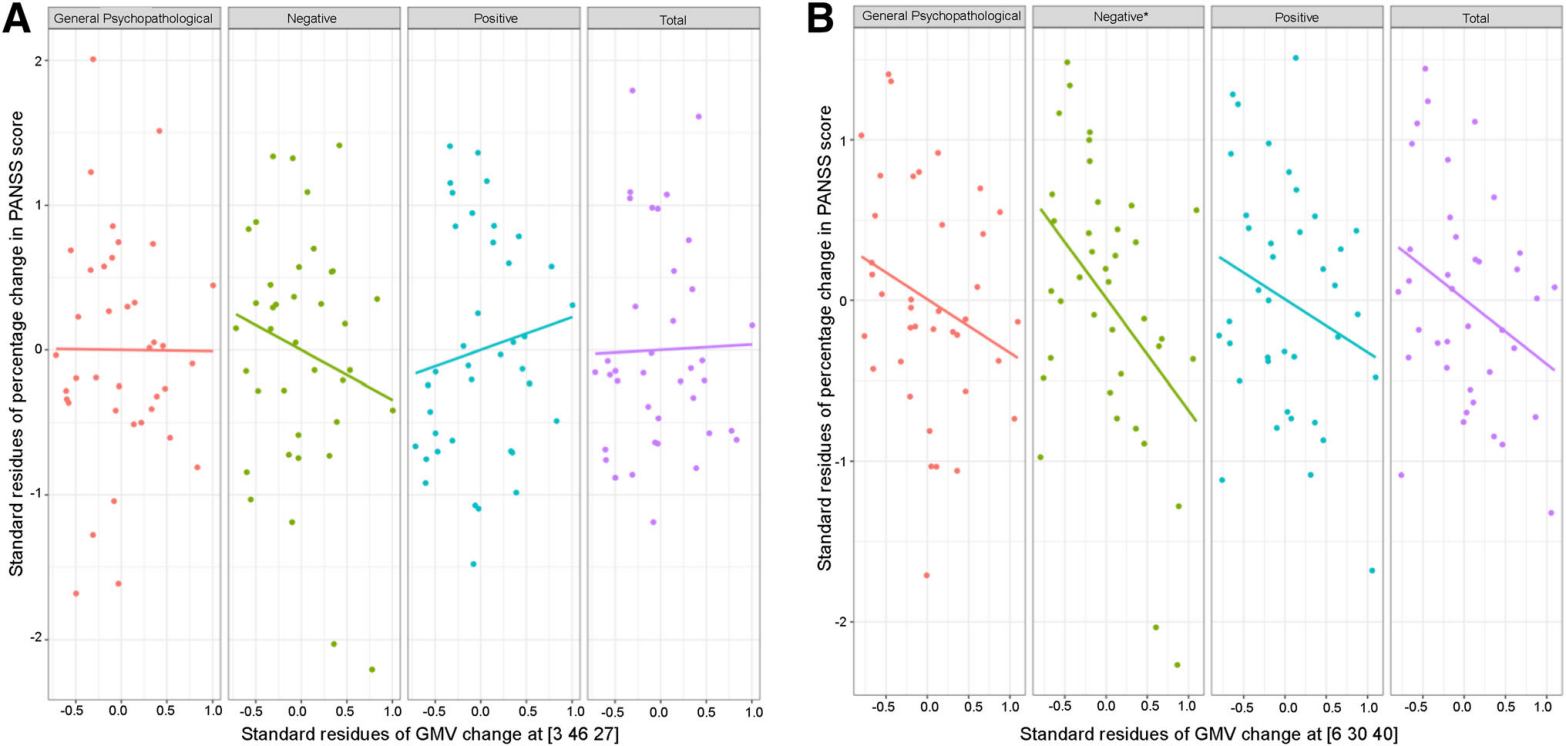
Relationships between longitudinal regional GMV reduction and the percentage changes of PANSS scores in the multilinear regression model after controlling for potential variables. **A** Longitudinal regional GMV reduction at the anterior rMFC [3 46 27] did not significantly influence the percentage changes of PANSS scores. **B** Longitudinal regional GMV reduction at the posterior rMFC [6 30 40] negatively influenced the percentage change of negative PANSS score.

Regional regression analysis (Table 3) showed that the baseline peak volumes at the anterior rMFC (regression coefficient = 0.621, *P *= 0.022) and posterior rMFC (regression coefficient = 0.924, *P* = 0.001) were both associated with a larger change in the positive PANSS score. Meanwhile, the posterior rMFC had an additional effect on the improvement of the general psychopathological (regression coefficient = 0.720, *P* = 0.006) and total PANSS scores (regression coefficient = 0.759, *P* = 0.002, Fig. 2). In the whole-brain voxel-wised regression analysis model conducted in SPM, we validated these correlations between baseline rMFC and rate of PANSS change (*P *< 0.001, uncorrected, Fig. 3). As for the influence of longitudinal GMV change, we found that the posterior rMFC shrinkage was negatively associated with improvement of the negative PANSS score (regression coefficient = −0.748 *P *= 0.019) (Fig. 4). We found that the baseline total GMV was negatively associated with an improvement in general psychopathological symptoms and the change in total GMV positively affected the improvement of negative PANSS scores only in the model of the posterior rMFC (Table 3).

## Discussion

Here, we investigated the longitudinal grey matter changes in patients with schizophrenia during short-term antipsychotic treatment, addressing the importance of GMV biomarkers for their relationship to antipsychotic efficacy. Longitudinally, there were progressive total and rMFC GMV decreases in the schizophrenia group after 6 weeks of antipsychotic treatment, while no significant changes were observed in the control group. Furthermore, a higher baseline rMFC GMV was correlated with better positive symptom improvement after treatment. In addition, a higher baseline posterior rMFC GMV predicted better remission of the general psychopathological symptoms, and a lesser reduction of GMV in this region predicted better remission of negative symptoms. These findings suggest that the baseline and progressive changes in the rMFC are potential biomarkers for the response to antipsychotic treatment. As for other variables, our analysis revealed that the treatment effect on total and negative symptoms was worsened by family history, and general psychopathological symptoms achieved more remission in patients with a shorter duration of illness and higher educational level.

The rMFC, one of the cortical midline structures, is a location for self-referential processing, constitutes the core of the self, and is critical for elaborating feelings of self [27, 28]. Besides, it has been proposed to be a site of cognitive control, including the detection of unfavorable outcomes, response conflict, and performance monitoring [29]. Task-related neural activations have revealed that the posterior rMFC is activated by more cognitive tasks while the anterior rMFC is activated by more emotional tasks [30]. In patients with schizophrenia, self-reference is typically reduced as a disorder of the self in both resting-state [31] and task-related activities [32]. Reduced task-related suppression of the rMFC may contribute to disturbances of thought in schizophrenia and risk for the illness [32]. Relative to healthy participants, schizophrenic patients exhibit reduced GMV in the rMFC, and this reduction is associated with poor performance on emotion attribution to protagonists in social situations [33]. N-acetylaspartate reduction in the medial prefrontal cortex has also been reported following 8 weeks of risperidone treatment in first-episode drug-naïve patients with schizophrenia [34], suggesting the importance of this region in the pathological development of schizophrenia. A longitudinal GMV reduction in schizophrenia patients has been found in many frontal cortical regions [12, 36–38], and our finding of rMFC shrinkage contributes to current knowledge of the structural pathology in schizophrenia.

A core finding of our analysis was that the GMV at baseline in the rMFC predicted an improvement of positive symptoms after 6 weeks of treatment. Positive symptoms, including hallucinations and delusions, in which contact with reality is lost, are explained in terms of the ability to distinguish the source of internal experiences from external reality [39]. Reality-monitoring is related to structural variability within the para-cingulate region of the medial anterior prefrontal cortex in healthy individuals [40]. The aberrant salience hypothesis of psychosis suggests that the positive symptom of delusions in patients results from cognitive efforts to understand aberrantly salient experiences, whereas hallucinations are internal representations [41, 42]. Abnormally low activation of the rMFC has been reported in patients with schizophrenia, and after 80 h of training, improved reality monitoring is associated with increased rMFC activity [43, 44]. The rMFC regions exhibit significantly reduced gyrification in patients who experience hallucinations compared with those who do not [45], indicating that it is a core region for reality-monitoring, and this has been further validated in functional MRI meta-analysis [46]. Thus, our finding suggests that people with increased baseline rMFC GMV may preserve better neural function, and that is why they are capable of achieving greater clinical relief of positive symptoms associated with impaired reality-monitoring after treatment.

The posterior rMFC, or the dorsal MFC, is associated with self-referential and social cognition [47], which are unique cognitive deficits in schizophrenia [48]. This functional overlap would be expected if humans use their own experiences to infer the mental states of others, a basic postulate of simulation theory [49, 50]. Prominent negative symptoms affect ~40% of people with schizophrenia and are closely associated with functional outcomes [51]. A greater activation in the posterior rMFC has been associated with higher negative affectivity in healthy individuals [52]. The healthy siblings of patients with schizophrenia exhibit exaggerated resting-state functional activity in the midline areas of the anterior and posterior rMFC during self-referential processing [53]. Patients with schizophrenia show lower functional connectivity between the mid/posterior cingulate gyrus and the posterior rMFC in resting-state fMRI and exhibit increased activity in this region when inferring the intentions of others [54, 55]. All these findings suggest that pathological changes in this region have something to do with self-referential and social cognition. The correlation between improved general psychopathological symptoms and a higher baseline posterior rMFC GMV, together with improved negative symptoms and less GMV reduction in the posterior rMFC contributes to our understanding of the potential role and clinical importance of this region.

We also reported progressive total GMV reduction in patients with schizophrenia after 6 weeks of antipsychotic treatment; this may be a result of the volume decrease in the rMFC or other neuropathological changes. The relationship between total GMV and treatment effect in the posterior rMFC model also indicates their correlation in a way. The progression of schizophrenia is accompanied by altered cortical plasticity and functioning [56], and longitudinal GMV reduction has been found in many studies, especially in frontal cortical regions [12, 36–38]. As for other variables, our analysis revealed that the treatment effect on total and negative symptoms was worsened by family history. This is within our expectation, as a positive family history of schizophrenia predicts a poorer long-term outcome [57]. Besides, general psychopathological symptoms achieved more remission in patients with a shorter duration of illness and higher educational level. Our current finding is in accord with the clinical experience that patients with a shorter duration of illness benefit more from treatment. It has been reported that a shorter duration of untreated psychosis is associated with a greater response to antipsychotic treatment in first-episode schizophrenia [58]. Our results indicated that patients with a higher level of education achieved greater clinical improvement, which was predictable from the improvement in attenuated positive symptoms among individuals at high risk [59]. However, why different clinical features affect different clinical categories might need to be explored in greater detail in the future. As for the CPZ eq dose levels, it was within our expectation that this was not associated with the treatment effect, as the dose for each patient was delicately decided by experienced doctors after considering the disease severity of each patient and possible sensitivity to treatment based on the medical history. All these regression results might serve as references for clinicians to make decision and estimate the prognosis of individual patients.

This study, although shedding light on potential predictive factors for antipsychotic treatment with grey matter changes, still has several limitations. First, we did not divide participants into subgroups based on first-episode or relapse owing to the limited sample size, so our group-level analysis could not exclude the possibility that the observed pathological changes occur only in a subset of patients with schizophrenia. Second, considering that the types of drugs patients took were complex and difficult to classify, we did the regression analysis with daily CPZ eq doses as a predictive factor and did not include the types of antipsychotic drugs. We guaranteed single-drug treatment for the majority of the patients in our study, but different drugs may have varying treatment effects in relation to brain structure [22]. Third, although we tried to establish a causal link between the biological markers and the symptom improvement using the linear regression model, a definite relationship cannot be affirmed yet. Hence, our findings of predictive factors for symptom improvement need to be verified in another independent, specifically designed study. Fourth, many potential factors contributing to changes in the brain and the effects of antipsychotic treatment need to be determined. For example, whether the effects of antipsychotic treatment vary with the stage of illness or occur only when a certain threshold of daily or cumulative dose is reached.

In conclusion, the baseline GMV of the rMFC might help to predict positive symptom remission related to reality-monitoring. The association of the posterior rMFC GMV with general psychopathological and negative symptoms might relate to self-referential processing and social cognition. Having a family history, a longer duration of illness, and a lower level of education might be associated with a bad prognosis for different clinical domains. This preliminary clarification of structural brain abnormalities and antipsychotic medication allow a better understanding of the pathological mechanisms in schizophrenia and might be important for clinicians to make clinical decisions.

## References

[1] JA Salomon, Vos T, Hogan DR, Gagnon M, Naghavi M, Mokdad A (2012). Common values in assessing health outcomes from disease and injury: Disability weights measurement study for the Global Burden of Disease Study 2010. Lancet.

[2] Kahn RS, Sommer IE, Murray RM, Meyer-Lindenberg A, Weinberger DR, Cannon TD, *et al.* Schizophrenia. Nat Rev Dis Primers 2015: 15067. 10.1038/nrdp.2015.67 27189524

[3] Goldstein JM, Goodman JM, Seidman LJ, Kennedy DN, Makris N, Lee H (1999). Cortical abnormalities in schizophrenia identified by structural magnetic resonance imaging. Arch Gen Psychiatry.

[4] Shenton ME, Dickey CC, Frumin M, McCarley RW (2001). A review of MRI findings in schizophrenia. Schizophr Res.

[5] Torres US, Duran FL, Schaufelberger MS, Crippa JA, Louzã MR, Sallet PC (2016). Patterns of regional gray matter loss at different stages of schizophrenia: A multisite, cross-sectional VBM study in first-episode and chronic illness. Neuroimage.

[6] Zhang C, Wang Q, Ni P, Deng W, Li Y, Zhao L (2017). Differential cortical gray matter deficits in adolescent- and adult-onset first-episode treatment-naïve patients with schizophrenia. Sci Rep.

[7] Olabi B, Ellison-Wright I, McIntosh AM, Wood SJ, Bullmore E, Lawrie SM (2011). Are there progressive brain changes in schizophrenia? A meta-analysis of structural magnetic resonance imaging studies. Biol Psychiatry.

[8] Hulshoff Pol HE, Kahn RS (2007). What happens after the first episode? A review of progressive brain changes in chronically ill patients with schizophrenia. Schizophr Bull.

[9] Haijma SV, Van Haren N, Cahn W, Koolschijn PC, Hulshoff Pol HE, Kahn RS (2013). Brain volumes in schizophrenia: a meta-analysis in over 18 000 subjects. Schizophr Bull.

[10] Fusar-Poli P, Smieskova R, Kempton MJ, Ho BC, Andreasen NC, Borgwardt S (2013). Progressive brain changes in schizophrenia related to antipsychotic treatment? A meta-analysis of longitudinal MRI studies. Neurosci Biobehav Rev.

[11] Deng MY, McAlonan GM, Cheung C, Chiu CP, Law CW, Cheung V (2009). A naturalistic study of grey matter volume increase after early treatment in anti-psychotic naive, newly diagnosed schizophrenia. Psychopharmacology (Berl).

[12] Girgis RR, Diwadkar VA, Nutche JJ, Sweeney JA, Keshavan MS, Hardan AY (2006). Risperidone in first-episode psychosis: a longitudinal, exploratory voxel-based morphometric study. Schizophr Res.

[13] Liao J, Yan H, Liu Q, Yan J, Zhang L, Jiang S (2015). Reduced paralimbic system gray matter volume in schizophrenia: Correlations with clinical variables, symptomatology and cognitive function. J Psychiatr Res.

[14] Frascarelli M, Tognin S, Mirigliani A, Parente F, Buzzanca A, Torti MC (2015). Medial frontal gyrus alterations in schizophrenia: relationship with duration of illness and executive dysfunction. Psychiatry Res.

[15] Burke L, Androutsos C, Jogia J, Byrne P, Frangou S (2008). The Maudsley Early Onset Schizophrenia Study: the effect of age of onset and illness duration on fronto-parietal gray matter. Eur Psychiatry.

[16] Staal WG, Hulshoff Pol HE, Schnack HG, van Haren NE, Seifert N, Kahn RS (2001). Structural brain abnormalities in chronic schizophrenia at the extremes of the outcome spectrum. Am J Psychiatry.

[17] Quarantelli M, Palladino O, Prinster A, Schiavone V, Carotenuto B, Brunetti A (2014). Patients with poor response to antipsychotics have a more severe pattern of frontal atrophy: a voxel-based morphometry study of treatment resistance in schizophrenia. Biomed Res Int.

[18] Li M, Chen Z, Deng W, He Z, Wang Q, Jiang L (2012). Volume increases in putamen associated with positive symptom reduction in previously drug-naive schizophrenia after 6 weeks antipsychotic treatment. Psychol Med.

[19] Emsley R, Asmal L, du Plessis S, Chiliza B, Phahladira L, Kilian S (2017). Brain volume changes over the first year of treatment in schizophrenia: relationships to antipsychotic treatment. Psychol Med.

[20] Friedman L, Knutson L, Shurell M, Meltzer HY (1991). Prefrontal sulcal prominence is inversely related to response to clozapine in schizophrenia. Biol Psychiatry.

[21] Molina V, Reig S, Sarramea F, Sanz J, Francisco Artaloytia J, Luque R (2003). Anatomical and functional brain variables associated with clozapine response in treatment-resistant schizophrenia. Psychiatry Res.

[22] Arango C, Breier A, McMahon R, Carpenter WT, Buchanan RW (2003). The relationship of clozapine and haloperidol treatment response to prefrontal, hippocampal, and caudate brain volumes. Am J Psychiatry.

[23] Yan CG, Wang XD, Zuo XN, Zang YF (2016). DPABI: Data processing & analysis for (resting-state) brain imaging. Neuroinformatics.

[24] Ashburner J (2007). A fast diffeomorphic image registration algorithm. Neuroimage.

[25] Kurth F, Gaser C, Luders E (2015). A 12-step user guide for analyzing voxel-wise gray matter asymmetries in statistical parametric mapping (SPM). Nat Protoc.

[26] Boter H, Peuskens J, Libiger J, Fleischhacker WW, Davidson M, Galderisi S (2009). Effectiveness of antipsychotics in first-episode schizophrenia and schizophreniform disorder on response and remission: an open randomized clinical trial (EUFEST). Schizophr Res.

[27] Northoff G, Heinzel A, de Greck M, Bermpohl F, Dobrowolny H, Panksepp J (2006). Self-referential processing in our brain–a meta-analysis of imaging studies on the self. Neuroimage.

[28] Gusnard DA, Akbudak E, Shulman GL, Raichle ME (2001). Medial prefrontal cortex and self-referential mental activity: relation to a default mode of brain function. Proc Natl Acad Sci U S A.

[29] Ridderinkhof KR, Ullsperger M, Crone EA, Nieuwenhuis S (2004). The role of the medial frontal cortex in cognitive control. Science.

[30] Amodio DM, Frith CD (2006). Meeting of minds: the medial frontal cortex and social cognition. Nat Rev Neurosci.

[31] Kuhn S, Gallinat J (2013). Resting-state brain activity in schizophrenia and major depression: a quantitative meta-analysis. Schizophr Bull.

[32] Whitfield-Gabrieli S, Thermenos HW, Milanovic S, Tsuang MT, Faraone SV, McCarley RW (2009). Hyperactivity and hyperconnectivity of the default network in schizophrenia and in first-degree relatives of persons with schizophrenia. Proc Natl Acad Sci U S A.

[33] Yamada M, Hirao K, Namiki C, Hanakawa T, Fukuyama H, Hayashi T (2007). Social cognition and frontal lobe pathology in schizophrenia: A voxel-based morphometric study. Neuroimage.

[34] Zong X, Hu M, Li Z, Cao H, He Y, Liao Y (2015). N-acetylaspartate reduction in the medial prefrontal cortex following 8 weeks of risperidone treatment in first-episode drug-naive schizophrenia patients. Sci Rep.

[35] Torres US, Portela-Oliveira E, Borgwardt S, Busatto GF (2013). Structural brain changes associated with antipsychotic treatment in schizophrenia as revealed by voxel-based morphometric MRI: an activation likelihood estimation meta-analysis. BMC Psychiatry.

[36] Leung M, Cheung C, Yu K, Yip B, Sham P, Li Q (2011). Gray matter in first-episode schizophrenia before and after antipsychotic drug treatment. Anatomical likelihood estimation meta-analyses with sample size weighting. Schizophr Bull.

[37] Prasad KM, Sahni SD, Rohm BR, Keshavan MS (2005). Dorsolateral prefrontal cortex morphology and short-term outcome in first-episode schizophrenia. Psychiatry Res.

[38] Kasparek T, Prikryl R, Schwarz D, Kucerova H, Marecek R, Mikl M (2009). Gray matter morphology and the level of functioning in one-year follow-up of first-episode schizophrenia patients. Prog Neuropsychopharmacol Biol Psychiatry.

[39] Owen MJ, Sawa A, Mortensen PB (2016). Schizophrenia. Lancet.

[40] Buda M, Fornito A, Bergström ZM, Simons JS (2011). A specific brain structural basis for individual differences in reality monitoring. J Neurosci.

[41] Kapur S (2003). Psychosis as a state of aberrant salience: a framework linking biology, phenomenology, and pharmacology in schizophrenia. Am J Psychiatry.

[42] Pankow A, Katthagen T, Diner S, Deserno L, Boehme R, Kathmann N (2016). Aberrant salience is related to dysfunctional self-referential processing in psychosis. Schizophr Bull.

[43] Subramaniam K, Luks TL, Fisher M, Simpson GV, Nagarajan S, Vinogradov S (2012). Computerized cognitive training restores neural activity within the reality monitoring network in schizophrenia. Neuron.

[44] Vinogradov S, Luks TL, Schulman BJ, Simpson GV (2008). Deficit in a neural correlate of reality monitoring in schizophrenia patients. Cereb Cortex.

[45] Garrison JR, Fernyhough C, McCarthy-Jones S, Haggard M, Australian Schizophrenia Research B, Simons JS (2015). Paracingulate sulcus morphology is associated with hallucinations in the human brain. Nat Commun.

[46] Zmigrod L, Garrison JR, Carr J, Simons JS (2016). The neural mechanisms of hallucinations: A quantitative meta-analysis of neuroimaging studies. Neurosci Biobehav Rev.

[47] Green MF, Leitman DI (2008). Social cognition in schizophrenia. Schizophr Bull.

[48] Fisher M, McCoy K, Poole JH, Vinogradov S (2008). Self and other in schizophrenia: A cognitive neuroscience perspective. Am J Psychiatry.

[49] Mitchell JP, Banaji MR, Macrae CN (2005). The link between social cognition and self-referential thought in the medial prefrontal cortex. J Cogn Neurosci.

[50] Benoit RG, Gilbert SJ, Volle E, Burgess PW (2010). When I think about me and simulate you: medial rostral prefrontal cortex and self-referential processes. Neuroimage.

[51] Carbon M, Correll CU (2014). Thinking and acting beyond the positive: the role of the cognitive and negative symptoms in schizophrenia. CNS Spectrums.

[52] Lemogne C, Gorwood P, Bergouignan L, Pélissolo A, Lehéricy S, Fossati P (2011). Negative affectivity, self-referential processing and the cortical midline structures. Soc Cogn Affect Neurosci.

[53] van Buuren M, Vink M, Kahn RS (2012). Default-mode network dysfunction and self-referential processing in healthy siblings of schizophrenia patients. Schizophr Res.

[54] Holt DJ, Cassidy BS, Andrews-Hanna JR, Lee SM, Coombs G, Goff DC (2011). An anterior-to-posterior shift in midline cortical activity in schizophrenia during self-reflection. Biol Psychiatry.

[55] Brüne M, Lissek S, Fuchs N, Witthaus H, Peters S, Nicolas V (2008). An fMRI study of theory of mind in schizophrenic patients with “passivity” symptoms. Neuropsychologia.

[56] Zhou D, Pang F, Liu S, Shen Y, Liu L, Fang Z (2017). Altered motor-striatal plasticity and cortical functioning in patients with schizophrenia. Neurosci Bull.

[57] Ran MS, Xiao Y, Zhao X, Zhang TM, Yu YH, Mao WJ (2017). Family history of psychosis and outcome of people with schizophrenia in rural China: 14-year follow-up study. Asian J Psychiatr.

[58] Perkins DO, Gu H, Boteva K, Lieberman JA (2005). Relationship between duration of untreated psychosis and outcome in first-episode schizophrenia: a critical review and meta-analysis. Am J Psychiatry.

[59] Kim M, Lee TH, Yoon YB, Lee TY, Kwon JS (2017). Predicting remission in subjects at clinical high risk for psychosis using mismatch negativity. Schizophr Bull.

